# A new approach for the analysis of facial growth and age estimation: Iris ratio

**DOI:** 10.1371/journal.pone.0180330

**Published:** 2017-07-07

**Authors:** Carlos Eduardo Palhares Machado, Marta Regina Pinheiro Flores, Laíse Nascimento Correia Lima, Rachel Lima Ribeiro Tinoco, Ademir Franco, Ana Cristina Barreto Bezerra, Martin Paul Evison, Marco Aurélio Guimarães

**Affiliations:** 1 National Institute of Criminalistics, Brazilian Federal Police, Ministry of Justice, Brasília, Distrito Federal, Brazil; 2 Health Science College, University of Brasília, Brasília, Distrito Federal, Brazil; 3 Medico Legal Centre, Department of Pathology and Legal Medicine, Ribeirão Preto Medical School, University of São Paulo, Ribeirão Preto, São Paulo, Brazil; 4 School of Dentistry, University of São Paulo, São Paulo, São Paulo, Brazil; 5 School of Dentistry, Federal University of Maranhão, São Luís, Maranhão, Brazil; 6 Piracicaba Dentistry School, State University of Campinas, Piracicaba, São Paulo, Brazil; 7 School of Dentistry, University Salgado de Oliveira, Niterói, Rio de Janeiro, Brazil; 8 Department of Anthropology, National Museum, Federal University of Rio de Janeiro, Rio de Janeiro, Rio de Janeiro, Brazil; 9 Department of Oral Health Sciences, Katholieke Universiteit, Leuven, Belgium; 10 Department of Applied Sciences, Centre for Forensic Science, Northumbria University, Newcastle, United Kingdom; Augusta University, UNITED STATES

## Abstract

The study of facial growth is explored in many fields of science, including anatomy, genetics, and forensics. In the field of forensics, it acts as a valuable tool for combating child pornography. The present research proposes a new method, based on relative measurements and fixed references of the human face—specifically considering measurements of the diameter of the iris (iris ratio)—for the analysis of facial growth in association with age in children and sub-adults. The experimental sample consisted of digital photographs of 1000 Brazilian subjects, aged between 6 and 22 years, distributed equally by sex and divided into five specific age groups (6, 10, 14, 18, and 22 year olds ± one month). The software package SAFF-2D^®^ (Forensic Facial Analysis System, Brazilian Federal Police, Brazil) was used for positioning 11 landmarks on the images. Ten measurements were calculated and used as fixed references to evaluate the growth of the other measurements for each age group, as well the accumulated growth (6–22 years old). The Intraclass Correlation Coefficient (ICC) was applied for the evaluation of intra-examiner and inter-examiner reliability within a specific set of images. Pearson’s Correlation Coefficient was used to assess the association between each measurement taken and the respective age groups. ANOVA and Post-hoc Tukey tests were used to search for statistical differences between the age groups. The outcomes indicated that facial structures grow with different timing in children and adolescents. Moreover, the growth allometry expressed in this study may be used to understand what structures have more or less proportional variation in function for the age ranges studied. The diameter of the iris was found to be the most stable measurement compared to the others and represented the best cephalometric measurement as a fixed reference for facial growth ratios (or indices). The method described shows promising potential for forensic applications, especially as part of the armamentarium against crimes involving child pornography and child abuse.

## Introduction

The human face does not grow homogenously over time. Each of the many facial structures develops in different dimensions and directions [1, 2]. Consequently, the facial anatomy reaches different proportions depending on age [1, 3, 4]. This phenomenon, known as allometry, is the reason why a child’s face does not correspond to a smaller version of an adult’s face. Growth and alterations in craniofacial morphology arouses interest in many fields of science, especially physical anthropology [5–7] and genetics [8]. In these fields, morphology is most often studied in relation to evolutionary process [9, 10] and medical therapeutics [11–15]. However, nowadays, alternative applications of craniofacial morphology, such as in the scope of forensic sciences, has received major attention. In this regard, forensic studies were developed in the last decade to investigate facial growth as an anthropometric tool for age estimation procedures in cases involving child pornography [16, 17].

Traditional anthropometry is performed by taking measurements directly from the subjects using calipers or measuring tapes. Clearly, this requires a controlled environment as well as the consent and cooperation of the examined subject—which can be a challenging task when children are involved [18]. Analysis of two-dimensional (2D) images, such as cephalograms and photographs, has consequently emerged as an alternative method for investigations in this field. Among the 2D techniques used for image acquisition and analysis of the human face, photo-anthropometry remains a popular approach for epidemiological and forensic studies. It consists of landmarking photographs to enable the measurement of distances, angles, and proportions [5]. The same principle is also applicable in three-dimensional (3D) images [15, 19].

A direct comparison between measurements taken from different photographs is only reliable if the images were acquired standardly or with metric references. In general, photographs could hamper anthropometric analyses. However, anthropometric exams are feasible if the photographs are taken following the same protocol. In this context, ratios and angles between facial distances could be calculated to allow an anthropometric exam, even in the absence of metric references in the image [20]. Yet, the ratios obtained between two linear facial distances culminate in indices that may play an import part in anthropology as tools for the classification of facial types [21].

In general, understanding the growth and alterations in craniofacial morphology requires long-term longitudinal studies that register human development from childhood to adulthood with direct or indirect measurements of the face. Using an innovative approach, Ferrario et al. [15] conducted a mixed longitudinal and cross-sectional study on the quantification of growth alterations in craniofacial morphology by employing a noninvasive 3D assessment that used cameras and infra-red sensors. Differences in growth timing and facial proportions between males and females could be detected following this approach. Other authors have similarly found other anthropological applications for the assessment of growth alterations in craniofacial morphology, namely the age estimation of children involved in pornography [17].

On one hand, allometry enables age estimation in forensic sciences. On the other hand, it limits the usefulness of facial indices for the detection of differences in growth proportions. This is justified since the indices are founded on two measurements that are taken from facial structures that develop within different timings. In other words, the use of indices to assess growth proportions is similar to estimating someone else’s car speed while driving a separate car. In this context, the outcome is not the real car speed, but rather the relative speed between both cars. For the human face, the use of indices results in relative variations of facial structures [2]. Despite their relative nature, measurements taken from the face are closer to real-life measurements if more stable facial structures are considered as references in the index equation.

The present study therefore aimed to analyze the allometry of human growth using a set of 10 facial measurements taken from children, adolescents, and young adults. The diameter of the iris was used as reference to assess the craniofacial variations in the remaining nine measurements.

## Material and methods

### Sampling and facial analysis

The sample consisted of 1000 photographs, taken standardly, of Brazilian subjects aged between 6 and 22 years. The sample was homogeneously divided (n = 200) into five age groups of 6, 10, 14, 18, and 22 year olds, with a standard deviation of 1 month in each age group. The photographs were selected from a database of the Brazilian Federal Police. For inclusion in the database, the photographs were taken following the International Civil Aviation Organization (ICAO) guidelines for passports and were stored in.PNG 24-bit format with resolution of 640×480 pixels. In addition, the same flash system and camera model were used for all photographs, and positioned 1.5 meters from the subject’s face. Only photographs of subjects with neutral facial expression, closed lips, and head positioned straight towards the camera were selected. Photographs of subjects with head rotation in the sagittal, axial, or coronal axes were excluded, as well as those with facial deformations or evident asymmetries. Subjects with facial hair, adornments, and make up were also excluded due to incomplete visualization of the face.

A cephalometric analysis of all photographs was performed by a single examiner. To assess intra-examiner reliability, a set of 100 images were examined in duplicate by positioning 11 landmarks on pre-established reference points (Table 1) [22]. At this step, a non-commercial software package developed by the Brazilian Federal Police for 2D facial analysis (SAFF-2D^®^–Forensic Facial Analysis System, Department of Federal Police, Brazil) was used. The software registers Cartesian coordinates in the x- and y-axes for each of the positioned landmarks. The intra- and inter-examiner reliability of the landmarking methodology was also assessed prior to the study and consisted of the analysis of 10 photographs by three trained examiners, three times, within an interval of 15 days.

**Table 1:** Definition of the cephalometric landmarks used in this study [2, 22].

Landmark	Definition
**1. Nasion (n)**	The interception of the midsagittal plane and the line crossing the superior palpebral creases, above the upper eyelids.
**2. Subnasale (sn)**	The lowest point of the nose on the midsagittal plane.
**3. Gnathion (gn)**	The lowest point of the chin, on the midsagittal plane.
**4. Endocanthion (en)**	The medial limit of the eye.
**5. Ectocanthion (ec)**	The lateral limit of the eye.
**6. Iridion laterale (il)**	The most lateral point of the rim of the iris.
**7. Iridion mediale (im)**	The most medial point of the rim of the iris.
**8. Pupil (pu)**^a^	The central point of the iris, mathematically calculated, between the Iridion laterale and the Iridion mediale of each eye.
**9. Zygion (zy)**	The widest point in the region of the zygomatic bone seen in the frontal view.
**10. Chelion (ch)**	The lateral limit of the mouth.
**11. Alare (al)**	The most lateral point on the “wing” of the nose.

^a^Not described, but calculated as the arithmetic mean between the Iridions.

### Calculation of absolute measurements

**Fig 1:**
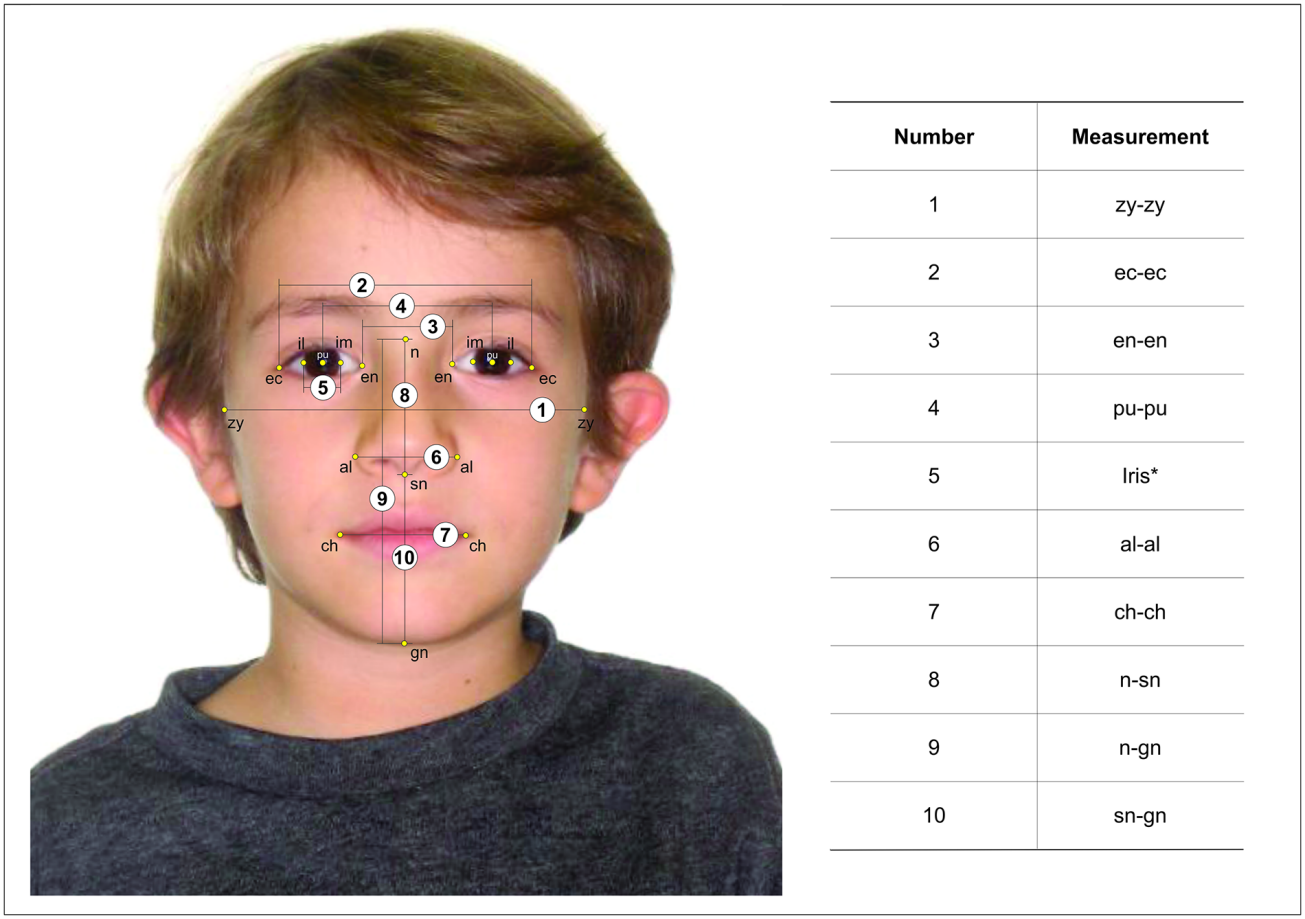
Graphic representation of the measurements adopted in this study. *The iris measurement corresponded to the average value of the right and left im-il; the image was obtained and illustratively used with the consent of the subject and relatives.

Once landmarks were registered, their coordinates were used to calculate 10 measurements expressed in pixels. Among the 10 measurements, nine were considered to be justified for the present study on the basis that they are commonly used to build facial indices in anthropometric studies [17, 18, 21, 22]. The remaining measurement calculated the diameter of the iris and was included as a new approach for anthropometric facial analysis (Fig 1).

### Relative Measurement Groups (RMG)

In order to enable the investigation of allometry among the 10 measurements, Relative Measurement Groups (RMG) were established (Table 2). These groups allowed the observation of growing differences from 10 diverse perspectives. This approach was founded on determining a fixed reference measurement to build specific ratios (similar to indices, but without expressing centesimal values) for each group. Variations between measurements were observed as a function of age.

**Table 2:** Relative Measurement Groups (RMG) and fixed measurements.

RMG	Fixed Measurement	n
RMG 1	zy-zy	1000
RMG 2	ec-ec	1000
RMG 3	en-en	1000
RMG 4	pu-pu	1000
RMG 5	iris*	1000
RMG 6	al-al	1000
RMG 7	ch-ch	1000
RMG 8	n-sn	1000
RMG 9	n-gn	1000
RMG 10	sn-gn	1000

*The iris was calculated by the arithmetic mean of the distances between the points il and im for each eye.

Since this approach is founded on the analysis of proportions, the fixed reference value could be selected randomly for each group. However, the mean values for the reference measurements from the total sample (n = 1000) were used to reach values closer to the actual facial measurements. Once the reference values were established, the images were scaled based on the percentage difference between the reference value and a target value for each group (size normalization); e.g. if the original reference measurement required enlargement by a factor of 1.5 to reach the target value, the same enlargement factor was applied to the remaining measurements. This procedure kept the metric proportions of the photographs unaltered and enabled the comparison of different facial structures from the same perspective in each RMG.

After establishing the RMG, the variables in each group were converted to percentage values (anthropometric ratios) of the reference measurements (Table 3). At this stage, the sample stratification by age range (6, 10, 14, 18, and 22 years old) was used to assess anthropometric variations during ageing. Despite knowing of the differences in craniofacial development between sexes [1], the present study did not aim to investigate craniofacial development in function of sex dimorphism. For that reason, the sample was analyzed grouped by sex.

**Table 3:** Representative formulas for calculating the proportional measurements for each Relative Measurement Group after image scaling.

RMG^a^	Fixed Measurement^b^ (n = 1000)	Proportional Measurements^c^ (Anthropometric Ratios)				
		1: zyzy	2: ecec	3: enen	…	10: sngn
1	$\bar{zyzy}$	$zyzy_{RMG1}=\frac{zyzy}{\bar{zyzy}}$	$ecec_{RMG1}=\frac{ecec}{\bar{zyzy}}$	$enen_{RMG1}=\frac{enen}{\bar{zyzy}}$	…	$sngn_{RMG1}=\frac{sngn}{\bar{zyzy}}$
2	$\bar{ecec}$	$zyzy_{RMG2}=\frac{zyzy}{\bar{ecec}}$	$ecec_{RMG2}=\frac{ecec}{\bar{ecec}}$	$enen_{RMG2}=\frac{enen}{\bar{ecec}}$	…	$sngn_{RMG2}=\frac{sngn}{\bar{ecec}}$
3	$\bar{enen}$	$zyzy_{RMG3}=\frac{zyzy}{\bar{enen}}$	$ecec_{RMG3}=\frac{ecec}{\bar{enen}}$	$enen_{RMG3}=\frac{enen}{\bar{enen}}$	…	$sngn_{RMG3}=\frac{sngn}{\bar{enen}}$
	…	…	…	…	…	…
10	$\bar{sngn}$	$zyzy_{RMG10}=\frac{zyzy}{\bar{sngn}}$	$ecec_{RMG10}=\frac{ecec}{\bar{sngn}}$	$enen_{RMG10}=\frac{enen}{\bar{sngn}}$	…	$sngn_{RMG10}=\frac{sngn}{\bar{sngn}}$

^a^**RMG**: Relative Measurement Group;

^b^**Fixed Measurement**: reference measurement used for calculating ratios obtained through the mean reference values of each group;

^c^**Proportional Measurements**: measurement calculated for each image from the ratio between the image measurement found and the mean value of the fixed (reference) measurement in each group (calculated for each image and each RMG).

### Calculation of Relative Growth (RG) and Average Relative Growth (ARG)

The Relative Growth (RG) was assessed for each age group, with the age of 6 years considered as the “zero point”, from which percentage variations were investigated as a function of age in relation to the other age subgroups. Once RG was obtained for each measurement, Average Relative Growth (ARG) was assessed by taking into consideration the values calculated for each RMG.

Table 4 details the process followed to quantify the ARG when using the craniofacial measurement 1 (zyzy) as reference. Analysis of the columns allows for the visualization of the proportional growth of the zyzy measurement within each of the 10 RMGs, in each of the different age ranges. Zero values in the first column are explained by the scaling process, which made the reference measurement fixed for all age ranges in RMG1 (zy-zy). The codification $\Delta \%\;zyzy_{6-10}^{RMG2}$ can be interpreted as the percentage variation of measurement zy-zy, within RMG2, when considering the mean values obtained from the cohort of 6 to 10 year olds (6–10); the column on the far right shows the ARG for the zy-zy measurement in each age range obtained from the mean values of the relative zy-zy growth, by considering all RMGs (i.e. $ARG_{6-10}^{zyzy}$, refers to the age range of 6–10 years). The bottom line of Table 4 shows the cumulative relative growth of zy-zy from 6 to 22 years of age.

**Table 4:** Quantification of the metric variation of measurement 1 (zy-zy) as a function of age and Relative Measurement Group to determine the Average Relative Growth.

Measurement	Age (years)	RMG1 zy-zy	RMG2 ec-ec	RMG3 en-en	…	RMG10: sn-gn	ARG
**1. zy-zy**	**6 to 10**	0	$\Delta \%\;{\text{zyzy}}_{6-10}^{\text{RMG2}}$	$\Delta \%\;{\text{zyzy}}_{6-10}^{\text{RMG}3}$	…	$\Delta \%\;{\text{zyzy}}_{6-10}^{\text{RMG}10}$	${\text{ARG}}_{6-10}^{\text{zyzy}}$
	**10 to 14**	0	$\Delta \%\;{\text{zyzy}}_{10-14}^{\text{RMG}2}$	$\Delta \%\;{\text{zyzy}}_{10-14}^{\text{RMG3}}$	…	$\Delta \%\;{\text{zyzy}}_{10-14}^{\text{RMG}10}$	${\text{ARG}}_{10-14}^{\text{zyzy}}$
	**14 to 18**	0	$\Delta \%\;{\text{zyzy}}_{14-18}^{\text{RMG}2}$	$\Delta \%\;{\text{zyzy}}_{14-18}^{\text{RMG}2}$	…	$\Delta \%\;{\text{zyzy}}_{14-18}^{\text{RMG}10}$	${\text{ARG}}_{14-18}^{\text{zyzy}}$
	**18 to 22**	0	$\Delta \%\;{\text{zyzy}}_{18-22}^{\text{RMG}2}$	$\Delta \%\;{\text{zyzy}}_{18-22}^{\text{RMG}2}$	…	$\Delta \%\;{\text{zyzy}}_{18-22}^{\text{RMG}10}$	${\text{ARG}}_{18-22}^{\text{zyzy}}$
	**6 to 22 (cumulative)**	**0**	$\Delta \%\;zyzy_{6-22}^{RMG2}$	$\Delta \%\;zyzy_{6-22}^{RMG3}$		$\Delta \%\;zyzy_{6-22}^{RMG10}$	$ARG_{18-22}^{zyzy}$

After ARG quantification for all measurements, a ranking of the relative variations was performed considering the age ranges separately and combined (cumulative growth). In this procedure, positive values indicated positive variations of a specific measurement compared to the mean growth of the others, while the opposite was interpreted for negative values.

The measurements that used iris diameter as reference (RMG5)–proposed as a new approach in the present study—were used as the parameter to analyze variation in the remaining measurements. Based on this analysis, a new ranking system was designed to provide outcomes closer to the real growth of the measurements considered.

### Statistical analysis

The Kolmogorov-Smirnov test was used to assess data normality and the Intraclass Correlation Coefficient (ICC) applied to the evaluation of the intra- and inter-examiner reliability for positioning landmarks and performing measurements. Descriptive statistics were used for initial data screening. Pearson’s Correlation Coefficient was used to associate measurements within each group and age. The statistical differences in each RMG were assessed by applying the ANOVA test as a function of age (considering the different age ranges). A Post-hoc Tukey test was used to search for statistical differences between specific age ranges and to verify the efficiency of each fixed measurement in detecting the relative growth.

All statistical tests were performed with the SPSS^®^ 21.0 software package (IBM^®^, New York, USA), with consideration for a significance level of 5% and confidence interval of 95%. Research databases used for stastical analysis may be found within the supporting information files S1–S6 Files.

### Ethics statement

The present research was conducted with the approval of the Committee of Ethics in Research of the University of São Paulo, under protocol number 17017213.0.0000.5440. The images used to illustrate the present study were acquired and used with the signed consent of the photographed subject and his relatives.

## Results

The data herein studied presented with a normal distribution. The Intraclass Correlation Coefficient revealed excellent outcomes for the landmarking performed in the intra- and inter-examiner reliability tests, which were performed prior to and during the research project (ICC > 0.90; p < 0.001). The mean and standard deviation of each of the cephalometric measurements obtained before size normalization are presented in Table 5. The values are expressed in pixels, with males and females grouped, considering the five age groups and the complete sample (6–22 years old).

**Table 5:** Cephalometric landmark measurements expressed in pixels.

Measurement	6 years (n = 200)		10 years (n = 200)		14 years (n = 200)		18 years (n = 200)		22 years (n = 200)		Total sample (n = 1000)	
	Mean	SD	Mean	SD	Mean	SD	Mean	SD	Mean	SD	Mean	SD
**1. zy-zy**	254.84	23.70	249.77	16.78	245.85	16.82	247.15	24.77	250.01	26.23	249.52	22.21
**2. ec-ec**	169.15	9.20	167.65	8.41	166.74	8.91	166.26	8.55	166.70	7.74	167.30	8.62
**3. en-en**	62.29	4.92	61.07	4.57	60.27	4.54	59.84	4.43	60.24	4.38	60.74	4.64
**4. pu-pu**	114.68	6.15	114.58	5.44	114.78	5.76	115.50	5.56	116.17	5.07	115.14	5.63
**5. iris**	24.96	1.63	23.58	1.64	22.20	1.67	21.67	1.64	21.56	1.45	22.79	2.07
**6. al-al**	64.86	4.74	65.04	5.69	67.56	6.00	68.04	5.79	67.81	5.85	66.66	5.80
**7. ch-ch**	80.10	8.29	83.98	7.77	85.27	8.22	85.94	8.35	87.32	6.64	84.52	8.24
**8. n-sn**	93.67	7.79	96.17	9.02	98.22	8.52	98.32	9.49	99.44	9.50	97.16	9.10
**9. n-gn**	205.58	13.77	211.15	13.93	216.44	15.92	219.91	15.76	223.73	15.40	215.36	16.27
**10. sn-gn**	111.97	10.35	115.03	10.07	118.27	11.19	121.64	10.90	124.36	13.76	118.25	12.15

The ARG of each measurement as a function of the age ranges are presented in Table 6. Using iris diameter, ARG reached the lowest values in all age groups in comparison to the other measurements. Positive values indicate a measurement with a relative growth higher than the mean variation of measurements, while negative values indicate the opposite. The iris values were consistently negative and was the measurement least altered with age. In the cumulative analysis (6–22 years), iris measurements reached a value 4.2 times lower (-14.83%) than the second highest ranked measurement (en-en, -4.62%).

**Table 6:** Average Relative Growth for the 10 measurements studied as a function of age.

Measurement	Average Relative Growth				
	6 to 10 years	10 to 14 years	14 to 18 years	18 to 22 years	6 to 22 years
**1. zy-zy**	-2.18%	-1.90%	0.23%	0.39%	**-3.29%**
**2. ec-ec**	-1.08%	-0.85%	-0.57%	-0.49%	**-2.80%**
**3. en-en**	-2.14%	-1.57%	-1.05%	-0.10%	**-4.62%**
**4. pu-pu**	-0.27%	-0.12%	0.33%	-0.17%	**-0.07%**
**5. Iris**	-5.74%	-6.20%	-2.62%	-1.23%	**-14.83%**
**6. al-al**	0.01%	3.52%	0.46%	-1.14%	**3.00%**
**7. ch-ch**	4.60%	1.23%	0.52%	0.87%	**7.54%**
**8. n-sn**	2.39%	1.84%	-0.22%	0.51%	**4.74%**
**9. n-gn**	2.49%	2.17%	1.31%	0.96%	**7.28%**
**10. sn-gn**	2.57%	2.45%	2.58%	1.33%	**9.41%**

**Fig 2:**
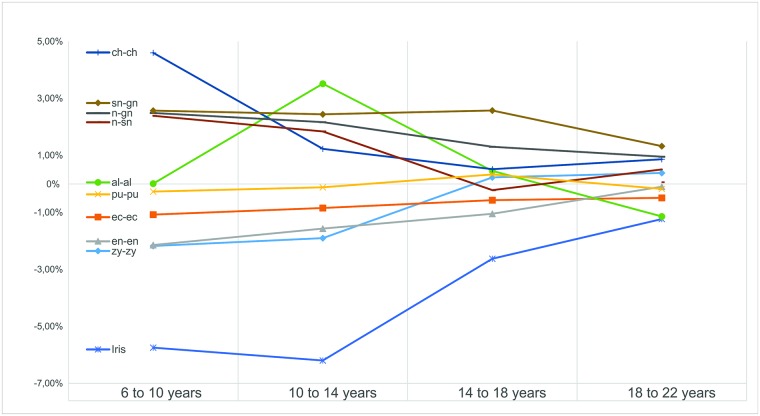
Average Relative Growth of the 10 measurements studied according to an age-segmental analysis.

**Fig 3:**
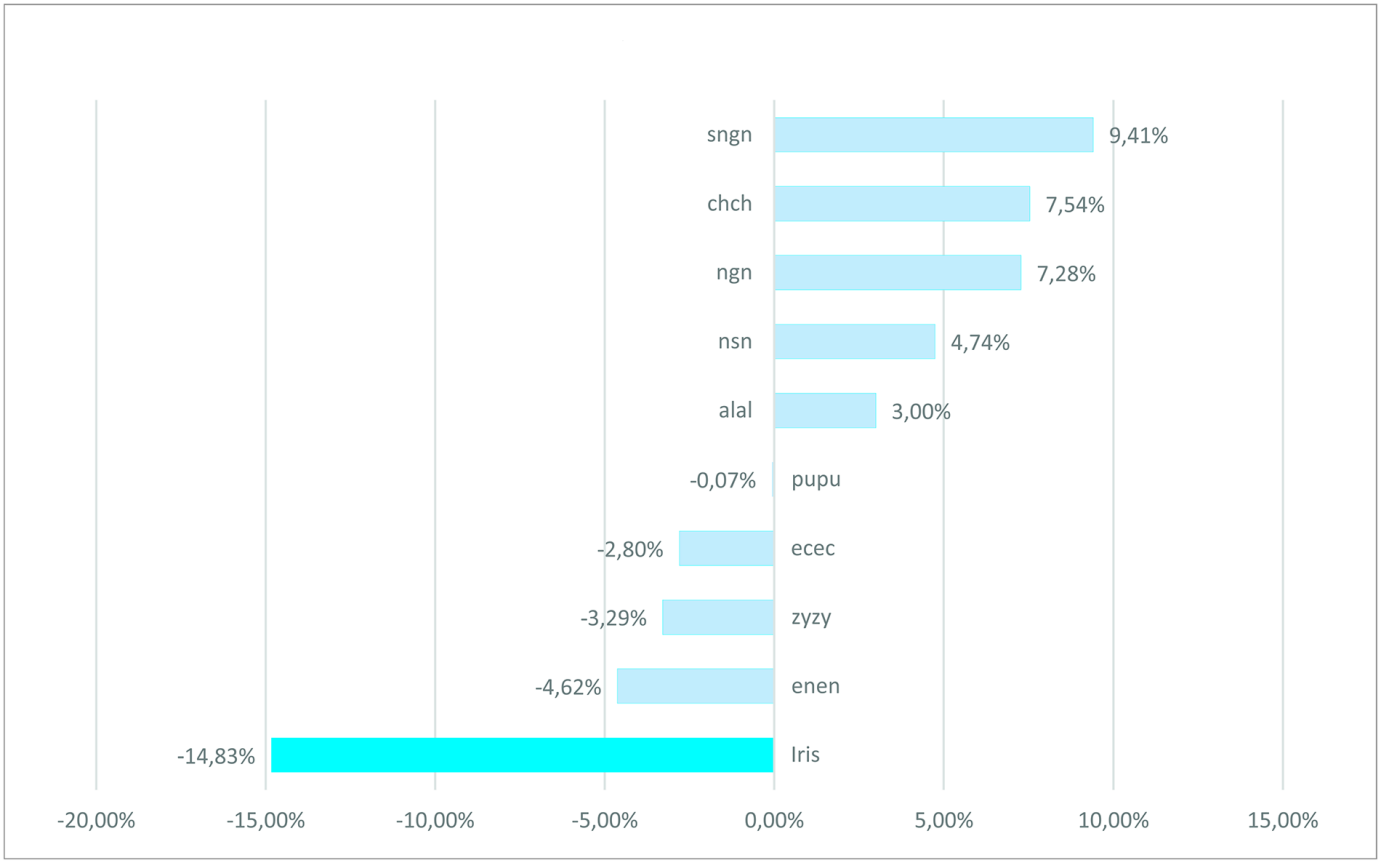
Ranking of the cumulative Average Relative Growth expressed as percentage values for the cumulative age range of 6 to 22 years.

Graphical representation of ARG progress between age groups (Fig 2) enables a clear interpretation of the data reported in Table 6, showing that facial measurements had different relative growth between the age groups. Some of the specific measurements presented ARG values that ranged between the positive and/or negative scale for the different age ranges (e.g. width of the face: zy-zy), while other measurements consistently remained positive or negative, such as the height of the lower third of the face (sn-gn) and the diameter of the iris. The cumulative approach for ARG (6–22 years old) enabled the construction of a proportional growth rank for each of the measurements (Fig 3). The height of the lower third of the face presented the highest positive value (+9.41%), indicating a greater relative growth, while the diameter of the iris had the most negative value (-14.83%), suggesting it is the most stable measurement of the study.

Table 7 expresses Pearson’s Correlation Coefficient (r) between age and the respective RMG measurements. Among the 81 possibilities expressed in the Table (9×9), only two combinations resulted in very strong correlation (0.8 ≤ |r| < 1) with age: the width of the lips (ch-ch) in RMG8 (n-sn), and the diameter of the iris in RMG9 (n-gn). The measurements reached outcomes that were more consistently and strongly correlated to age within RMG5 (Iris ratios), reporting seven values with strong correlation (0.6 ≤ |r| < 0.8) and two with moderate correlation (0.4 ≤ |r| < 0.6), among nine possibilities. Apart from these measurements, most combinations resulted in weak (0.2 ≤ |r| < 0.4) or very weak (0 < |r| < 0.2) correlations with age. Considering the mean values of the correlations (bottom line), it is possible to infer that all groups had a weak correlation with age, except for the group that used the diameter of the iris as reference (RMG5).

**Table 7:** Pearson’s Correlation Coefficient (r) assessment of the correlation between the 10 measurements and age (6–22 years) in relation to each RMG.

Measurement	Relative Measurement Group—Pearson’s Correlation Coefficient (r)									
	RMG1: zy-zy	RMG2: ec-ec	RMG3: en-en	RMG4: pu-pu	RMG5: Iris	RMG6: al-al	RMG7: ch-ch	RMG8: n-sn	RMG9: n-gn	RMG10: sn-gn
**1. zy-zy**	.^a^	-.024	.055	-.155**	.489**	-.265**	-.287**	-.261**	-.400**	-.298**
**2. ec-ec**	.047	.^a^	.109**	-.445**	.677**	-.320**	-.333**	-.327**	-.567**	-.318**
**3. en-en**	-.058	-.113**	.^a^	-.284**	.417**	-.323**	-.331**	-.290**	-.431**	-.320**
**4. pu-pu**	.219**	.445**	.281**	.^a^	.709**	-.186**	-.237**	-.185**	-.404**	-.234**
**5. Iris**	-.596**	-.682**	-.421**	-.713**	.^a^	-.645**	-.628**	-.696**	-.804**	-.567**
**6. al-al**	.310**	.317**	.323**	.189**	.629**	.^a^	-.085**	-.008	-.150**	-.142**
**7. ch-ch**	.356**	.376**	.343**	.262**	.672**	.104**	.^a^	.081*	-.019	-.053
**8. n-sn**	.303**	.324**	.286**	.189**	.685**	.017	-.067*	.^a^	-.132**	-.062
**9. n-gn**	.512**	.566**	.426**	.407**	.799**	.152**	.014	.149**	.^a^	-.062
**10. sn-gn**	.445**	.444**	.412**	.350**	.684**	.196**	.075*	.150**	.133**	.^a^
**MEAN**^b^	**0.32**	**0.37**	**0.30**	**0.33**	**0.64**	**0.25**	**0.23**	**0.24**	**0.34**	**0.23**

^a^. Not calculated since one or more variables are constant.

^b^. Mean calculated using absolute values.

**p < 0.01, level of significance;

*p < 0.05, level of significance

The ANOVA test indicated a statistically significant growth (p < 0.05) in all RMGs, for almost all the relative measurements, and showed metric differences from 6 to 22 years. On the other hand, the post-hoc test (Table 8) indicated that the differences in growth mainly took place in the two lower age ranges (6–10, 10–14 years old), while RG detection fell dramatically in the older age ranges (14–18, 18–22 years old). The RMG that employed the diameter of the iris as the reference measurement (RMG5) was the group with more measurements capable of detecting growth in the age ranges 6–10, 10–14, and 14–18 years. In the age range of 18–22 years, growth differences were only observed for seven measurement associations out of the 81 possibilities when considering all RMGs (Table 8).

**Table 8:** Post hoc Tukey test outcomes showing the statistical growth differences between each age range in relation to the different measurements of the Relative Measurement Groups (RMG).

Age (years)	Measurement	Relative Measurement Group									
		RMG1: zy-zy	RMG2: ec-ec	RMG3: en-en	RMG4: pu-pu	RMG5: Iris	RMG6: al-al	RMG7: ch-ch	RMG8: n-sn	RMG9: n-gn	RMG10: sn-gn
6 to 10	1. zy-zy					*		*	*	*	*
	2. ec-ec				*	*		*	*	*	*
	3. em-en				*	*		*	*	*	*
	4. pu-pu	*	*	*		*		*	*	*	
	5. Iris	*	*	*	*		*	*	*	*	*
	6. al-al					*		*		*	
	7. ch-ch	*	*	*	*	*	*				
	8. n-sn	*	*	*	*	*					
	9. n-gn	*	*	*	*	*	*				
	10. sn-gn	*	*	*	*	*					
10 to 14	1. zy-zy					*	*		*	*	*
	2. ec-ec				*	*	*		*	*	*
	3. em-en					*	*		*	*	*
	4. pu-pu	*	*			*	*			*	
	5. Iris	*	*	*	*		*	*	*	*	*
	6. al-al	*	*	*	*	*					
	7. ch-ch	*				*					
	8. n-sn	*	*	*		*					
	9. n-gn	*	*	*	*	*					
	10. sn-gn	*	*	*	*	*					
14 to 22	1. zy-zy					*					
	2. ec-ec				*	*				*	
	3. em-en										
	4. pu-pu		*			*					
	5. Iris	*	*		*		*		*	*	*
	6. al-al					*					
	7. ch-ch					*					
	8. n-sn					*					
	9. n-gn		*			*					
	10. sn-gn	*	*	*		*					
18 to 22	1. zy-zy										
	2. ec-ec									*	
	3. em-en										
	4. pu-pu										
	5. Iris									*	
	6. al-al									*	
	7. ch-ch										
	8. n-sn										
	9. n-gn		*			*	*				
	10. sn-gn					*					

*statistically significant differences (p < 0.05)

**Fig 4:**
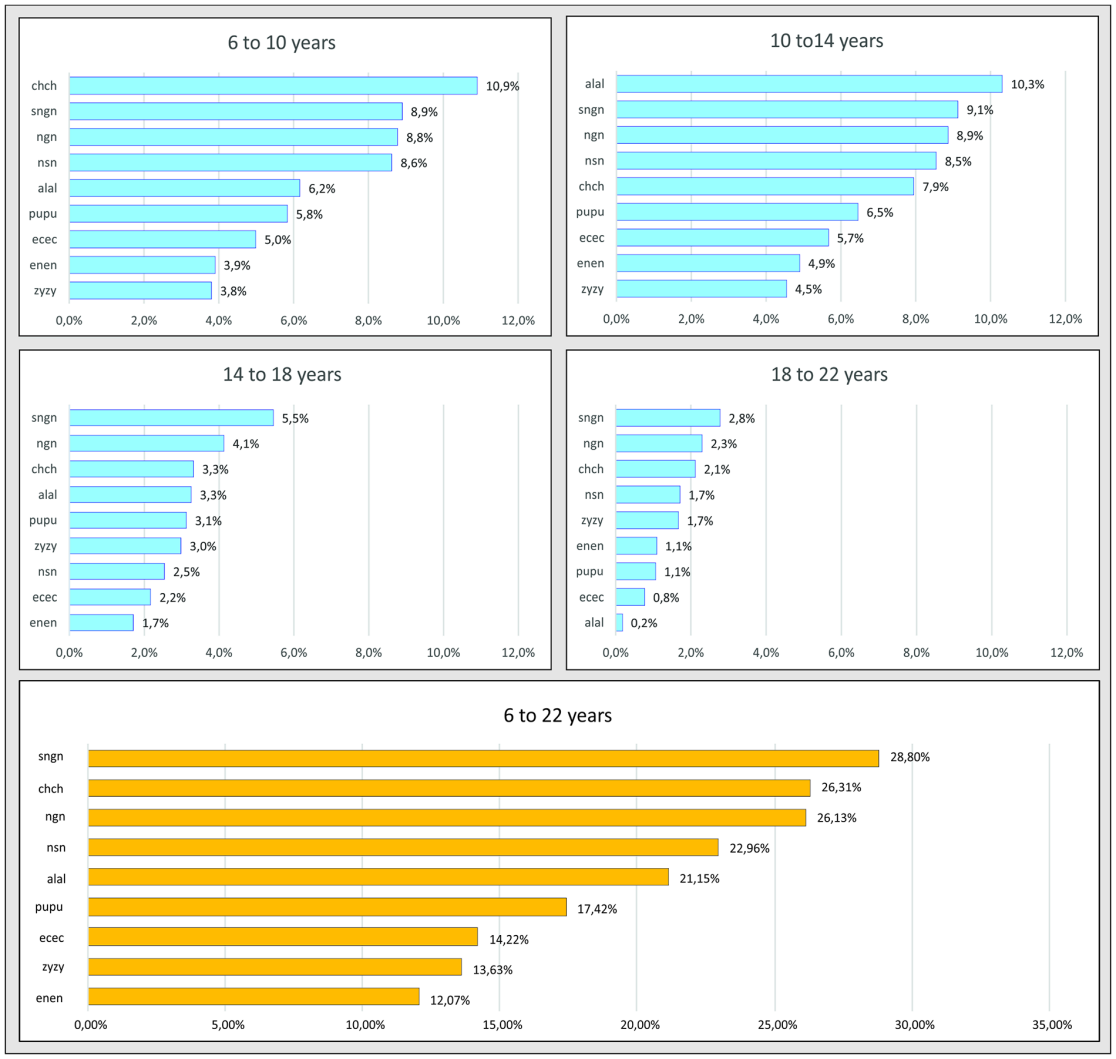
Facial growth ranking for the four age ranges (6–10, 10–14, 14–18, 18–22) and for cumulative growth (6–22) taking the diameter of the iris as the fixed reference.

Once the iris measurement was observed to offer the best outcome, it was used to evaluate facial growth with a more accurate perception of the metric changes that occur in the facial structures according to age (Fig 4). In subjects aged between 6–10 years, mouth width was the facial dimension with the highest growth percentage (10.9%). Yet in subjects aged 10–14 years, the nasal width presented highest growth percentage (10.3%), while in subjects aged 14–18 and 18–22 years, the height of the lower third of the face was the facial dimension with highest growth percentage—presenting 5.5% and 2.8% growth, respectively. The height of the lower third of the face also displayed the highest growth percentage (28.8%) in the cumulative analysis (6–22 years old). This growth percentage was more than twice that of face width (zy-zy: 13.63%).

## Discussion

Craniofacial growth is a continuous morphogenetic process that modifies a set of related anatomic structures in a morphological, functional, and balanced manner. This process is primarily explored in the fields of anthropology, genetics, dentistry, medicine, and forensics [1, 8]. Allometry, which is founded on the comparisons of anatomic proportions, is one of the mechanisms for examining the craniofacial growth process. Despite being more commonly used in comparative animal biology than in human anthropology [2], allometry recently emerged as a potential tool for forensic age estimation.

According to the Study Group on Forensic Age Diagnostics, standard parameters for age estimation are necessary to guide best practices internationally [23–24]. Special attention is given to ages of major legal importance, such as 14, 18, and 21 years [25–28]. As recommended by the Group, estimated age should be based on three independent evaluations, namely: 1) clinical examination using anthropological aspects and sexual maturity; 2) hand and wrist examination using carpal radiographs; and 3) dental examination using panoramic radiographs [29, 30]. However, under special circumstances, the only evidence available for analysis is a photograph, in which the abovementioned guidelines are not applicable. Analysis of the maturation of sexual traits may be considered when full body images are obtained; however, age estimation through an analysis of sexual maturation using photographs should not be considered for forensic purposes [17, 30]. This is primarily justified by the fact that outcomes may be biased by intentional modifications, such as the removal of pubic hair [16, 17]. This type of evidence is most common in cybercrimes involving child abuse. In these crimes, victims and perpetrators may be photographed [30] and their facial traits may contribute to crime characterization. Nevertheless, age estimation through facial photographs is a challenging procedure.

The search for age indicators through facial traits is justified by Cattaneo et al. [16]. The authors provided images of models (aged >18 years old) from a pornographic website to 23 examiners, consisting of forensic experts, pediatricians, gynecologists, and laypersons. The examiners were asked to estimate the age of the models mentioning which photographic indicator of age contributed most significantly in each case. Facial traits were described as the most cited indicator, reaching 64% of the forensic experts’ opinions. This outcome triggered further research in this field, including the development of methodologies based on facial indices for forensic age estimation [17].

Anthropometric indices represent a practical tool for analyzing the proportions of the human body. When used for forensic purposes, they may provide an indication of age, sex, and ancestry [21]. These indices have optimal application for the separate analysis of anthropometric measurements, such as the determination of sex using the ischium-pubic index. On the other hand, limitations may be found when the anthropometric analysis depends on a combined set of measurements, such as the evaluation of facial growth—in which several anatomic structures grow simultaneously and with different timings. The present study differs from the current literature [2, 5, 16–18] by suggesting the use of fixed measurements to verify the variation of all the other facial structures that surround it. This approach enables a comparison within and between different sets of anthropometric measurements by checking their variation according to age.

The results obtained using the present methodology should be analyzed with the understanding that facial structures do not decrease in size. More specifically, generation of negative values solely indicates that one anatomic structure presented a growth speed slower than the fixed reference measurement. Accordingly, a growth rate of 0.00% must not be translated as a lack of development, but rather as a proportional growth speed between two facial structures. This interpretation is most apparent when observing an ARG rate of 0.00% for nasal width (al-al) of the 6 to 10 year olds. It indicates that this anatomic structure grew in proportion with the average growth of all other facial structures. However, from 10 to 14 years old (equivalent to the growth spurt in the puberty period), the same structure (al-al) showed the highest average growth rate among all other structures. By contrast, the iris showed a considerably lower average growth rate in comparison to the other structures. This therefore represented the most stable structure in the present study, as indicated by the negative values for the iris’ RG rate in all the comparison groups.

Augusteyn et al. [31] addressed the stability of the iris’ dimensions through a growth analysis of eyeballs from patients (n = 541) aged from 0 to 104 years old. The authors observed that this structure has a predominant growth in the pre-natal stage, ending in the first year of life. Consequently, the iris trends to maintain its dimensions throughout life. Driessen et al. [23], 2011, also observed the stability on the dimensions of the iris. The authors designed a study with photo-anthropometry for the purposes of facial reconstruction. The diameter of the iris was tested based on its applicability as a calibration feature. Specifically, the iris was used as a fixed anatomic structure to register different photographs of the same person that underwent plastic surgery. Despite proving useful in these previous studies, the diameter of the iris is not commonly addressed in cephalometric analyses for anthropological purposes, especially for age estimation based on facial growth. One of the few studies that used the iris as a photo-anthropometric reference was made by George [22], which introduced the terms Iridion mediale and Iridion laterale as "*new proposals*" in the field of forensic facial analysis.

**Fig 5:**
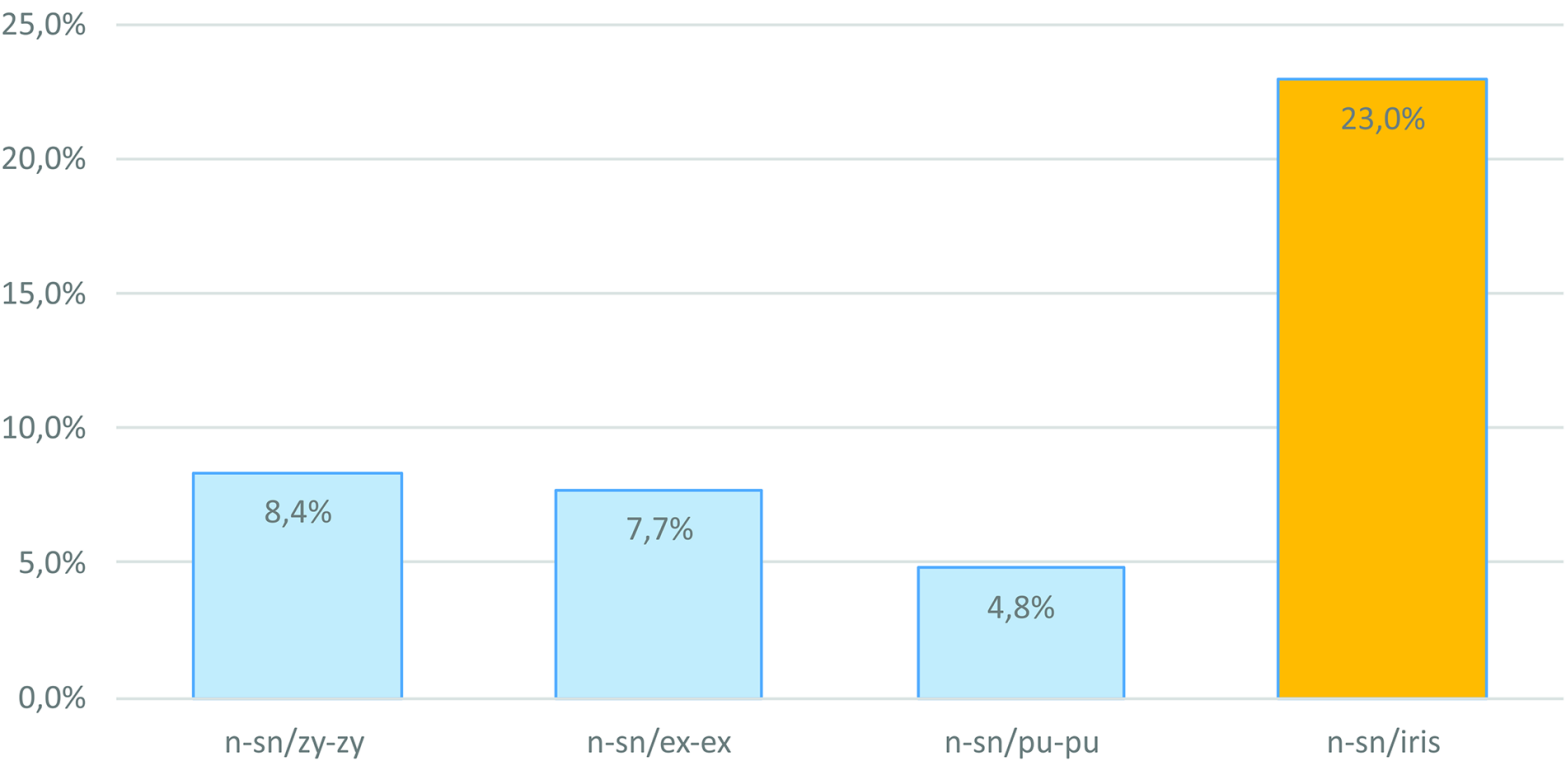
Comparison between the growth patterns of the height of the middle third of the face (n-sn) according to different fixed references. From the left to the right: zy-zy; ex-ex; pu-pu; and iris diameter.

The higher efficiency of iris diameter as a reference to detect metric changes in sub-adults becomes more evident when evaluating RG, by comparing the diameter of the iris with the other structures. Taking, as an example, the cumulative RG of nasal height (n-sn) (from 6 to 22 years old) and estimating growth using three indices (n-sn/zy-zy; n-sn/ex-ex; n-sn/pu-pu) found in the literature [17, 18], it is possible to reach the following growth rates: 8.4%, 7.7%, and 4.8%, respectively. When the three indices are replaced with measurements relative to the diameter of the iris (n-sn/iris), the growth rate increases to 23% (Fig 5). From a practical point of view, the diameter of the iris as a measurement reference (iris ratio) was more efficient n detecting the growth of nasal height, with results 4.8 times more accurate than interpupillary distance, which is more often used for facial metric analyses.

The results of this study indicate that the diameter of the iris may be considered as a promising reference metric for the analysis of facial images, especially for age estimation based on facial growth. The presented findings are even more relevant when considering that they were achieved with grouped sexes and within a population of high miscegenation (Brazilian). The strong correlation between iris measurement and age in the Brazilian population suggests the potential applicability of this method in a more homogeneous population, stratified by sex, would tend to be more discriminating for age ranges and facial structures.

In comparison to other studies that have investigated facial proportions for age estimation [17], the present study applied a different approach by combining a post-hoc Tukey test with ANOVA statistics. This enabled an analysis between age ranges (post-hoc Tukey test) and not only by grouping them (ANOVA). Interestingly, ANOVA statistics pointed toward growth differences in all groups, whilst the post-hoc Tukey test showed that only a small part of the statistical associations were efficient in detecting growth after the age of 14. However, since child abuse cases often involve victims younger than 14 years old, the positive outcomes from the present study in the lower age ranges may support the Courts with both technical and scientific evidence against these crimes.

Despite being more efficient for age estimation of subjects younger than 14, the proposed method still detected facial growth during post-pubertal stages. In fact, the results differed from traditional findings described in the literature [1]. Instead of detecting the end of facial growth around the age of 16 (post-pubertal age) [12–14, 32], the present study verified residual facial growth between the ages of 18 and 22. This growth was predominantly located in the height of the lower third of the face (sn-gn, with 5.5% in subjects aged 14–18 years old; and 2.8% in subjects aged 18–22 years old) and was found to be statistically significant according to the post-hoc Tukey test. These results also corroborate the findings that indicate early development of the neurocranium compared to the esplanchnocranium [1].

The major limitation of the method presented in this study concerns the need for facial photographs with a minimum resolution in the face area to enable the correct measurement of the diameter of the Iris and the consequent calculation of ratios. Unfortunately, these images are not commonly found during routine forensic investigations [33]. Despite using 2D images, the present study has potential application not only in 2D but also in 3D imaging, particularly in cases in which reconstructions are made using non-standardized imaging [19].

**Fig 6:**
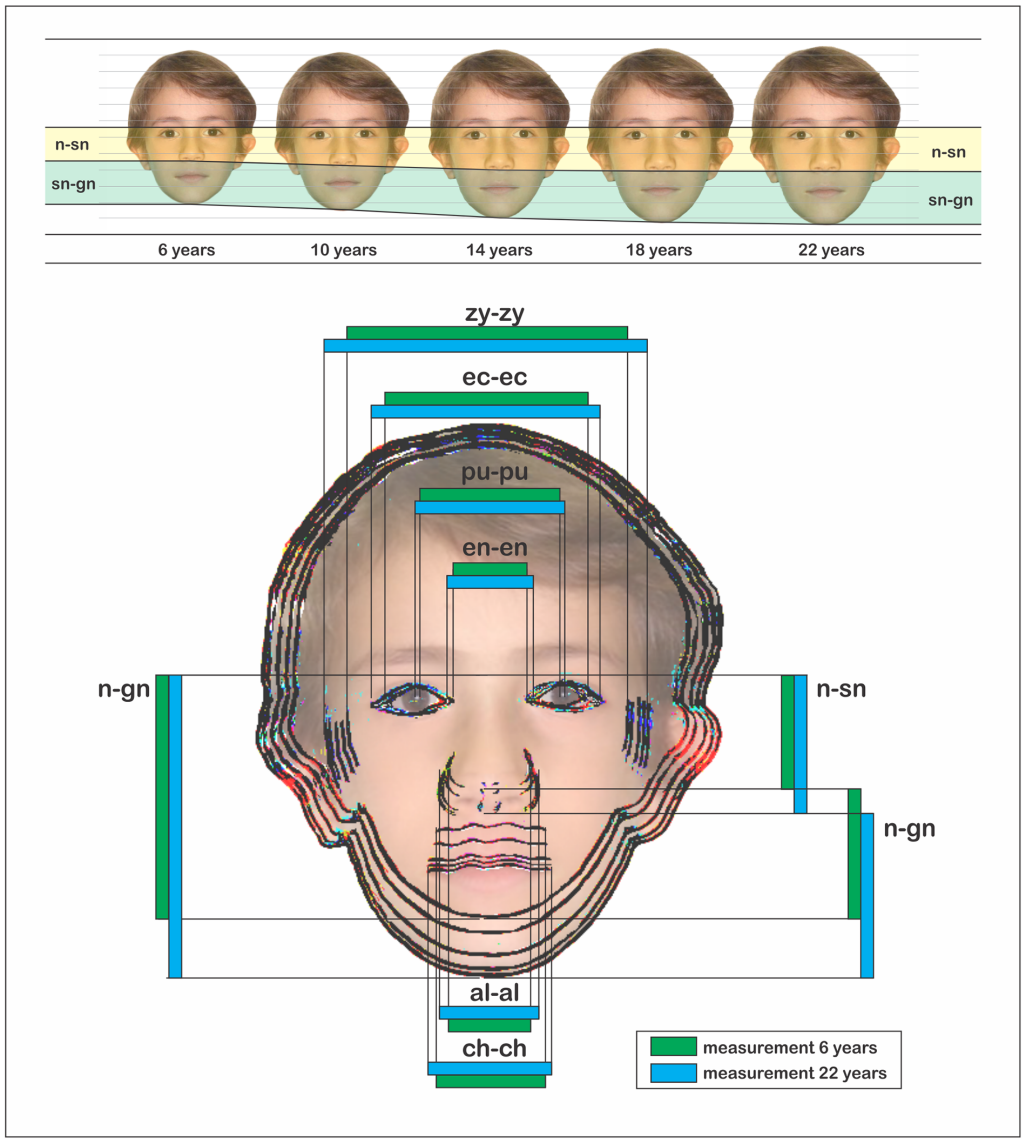
Facial progression based on age from a standard photograph of a 6-year-old boy (above). Superimposition with the projection of facial growth by considering the iris ratio for each measurement adopted in the study (below).

The facial growth pattern obtained using the iris RMG is visually expressed in Fig 6, in which different proportions and timings between the anatomic structures are illustrated (according to growth outcomes). Moreover, this images projects the growth pattern as a visual representation, translating the study results to a real world application of the present method in forensic practice—including age estimation and facial age progression. Both this applications play an important role by giving an indication of the age of the victims and suspects involved in child abuse cases, as well narrowing the search for missing children.

## Conclusion

The facial measurements quantified using fixed anthropometric references proved to be a potential tool for the photographic study of facial growth, representing an alternative to the conventional facial indices currently used in forensic investigations.

The diameter of the iris was the most efficient fixed anthropometric reference for the detection of facial growth. Application of this anthropometric reference in further studies is promising and brings innovative performance to forensic practice, especially with regard to age estimation of victims involved in child abuse cases.

Validation studies on the relative growth of anthropometric references using this newly described method are encouraged in different populations, as well in 3D images. Additionally, its application must be extrapolated to alternative applications in forensic anthropology, such as the determination of sex and ancestry, through facial patterns.

## Supporting information

S1 File Research database with the quantification of facial measurements. Quantification distributed on sex (M: males; F: females) and relative measurement group (RMG) for each of the ten measurements considered in the present study (M01-M10)
(XLSX)

S2 File Quantification of facial measurements performed to test intra-examiner agreement. Quantification distributed on sex (M: males; F: females) and age for each of the ten measurements considered in the present study (01–10)
(XLSX)

S3 File Quantification of facial measurements performed to test inter-examiner agreement. Quantification distributed on sex (M: males; F: females) and examiner for each of the ten measurements considered in the present study (from zy-zy to sn-gn)
(XLSX)

S4 File Facial measurements used to calculate ANOVA and Tukey statistic tests. Tests performed between groups (GC1-GC10) for each of the ten measurements considered in the present study (M01-M10)
(XLSX)

S5 File Research descriptive statistics. Descriptive data for the relative measurements groups (RMG), each of the ten (01–10) fixed facial measurements considered in the present study and their Average Relative Growth (ARG).
(XLSX)

S6 File Intraclass Correlation Coefficient (ICC) performed to tests statistically the intra- and inter-examiner agreement. Inter- and intra-examiner agreement quantified for each of the ten groups (CG1-CG10) considered in the present study.
(XLSX)
